# Changing the narrative: Shifting soft skills to power skills in nursing

**DOI:** 10.1097/nmg.0000000000000360

**Published:** 2026-04-28

**Authors:** Cynda Hylton Rushton, Mikhail Shneyder, Antonia M. Villarruel

**Affiliations:** **Cynda Hylton Rushton** is the Anne & George L. Bunting Professor of Clinical Ethics, Nursing & Pediatrics at Johns Hopkins University School of Nursing, Berman Institute of Bioethics in Baltimore, MD; **Mikhail Shneyder** is Founder and Chief Executive Officer of Nightingale Education Group in Salt Lake City, UT; and **Antonia M. Villarruel** is Professor and Margaret Bond Simon Dean of Nursing at the University of Pennsylvania School of Nursing in Philadelphia, PA.

**Keywords:** leadership, narrative, nursing, relational communication

## Abstract

Reframing nurses' core relational abilities from “soft skills” to “power skills” is essential to transforming health care. These capacities—such as effective communication, empathy, self-regulation, ethical grounding, and relational presence—aren't secondary to technical expertise but foundational to healing, trust, and professional identity. Power skills enable nurses to mitigate suffering, strengthen connections, and lead effectively across education, practice, and policy. This article calls on nurses in all roles to embrace, model, and structurally embed this narrative shift to reclaim nursing's authority, purpose, and enduring impact.

**Figure:**
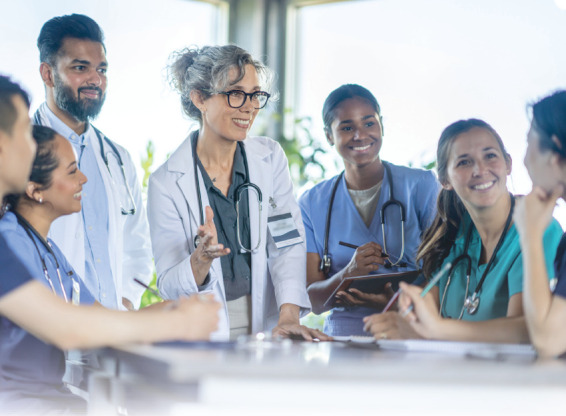
No caption available.

Relational care has always been the heartbeat of nursing—our ability to be present, to listen deeply, communicate clearly, and act with empathy and integrity. Too often, these vital capacities are labeled “soft skills,” mistakenly considered secondary to technical expertise. Yet they are the very skills that fuel authentic connection and understanding and positively impact health outcomes. The term, soft skills, originated with the US Army in the 1960s to refer to any skills that didn't relate to operating machinery but were recognized as pivotal to achieving positive outcomes and enhanced human performance in military exercises.^1^ These skills are essential in business, and health care leaders are implored to focus on them to manage the human and technical components of health care delivery.^2^ The COVID-19 pandemic exposed just how essential relational care is, not only for patients and families, but for nurses' well-being and the trust communities place in health care systems.

These skills aren't optional add-ons for nurses. They're foundational to nurses' ethical identity, central to healing, and indispensable to high-quality patient care. They reflect a cultivated set of capacities anchored in self-awareness, self-regulation, and skillful means. We refer to skills with the ability to transform relationships, mitigate suffering, and sustain trust within health care and society as “power skills.”

Rev. Dr. Martin Luther King said: “Power properly understood is nothing but the ability to achieve purpose. It is the strength required to bring about social, political, and economic change.”^3^ In this sense, power has neither a positive or negative valence. The kind of power we invoke here is neither coercive nor exploitative, nor is it exercised to control or dominate others. Rather, it's a form of power grounded in connection, empathy, and relational integrity. Understood in this way, power creates the conditions in which individuals can remain whole and undiminished while working in service of a greater good—one that emerges when people are respected, honored, and supported in exercising their agency.^4^ This form of power enables principled and courageous action without “powering over” others; instead of relying on shame, fear, or ego-driven competition, it draws strength from shared purpose, mutual recognition, and ethical commitment.

We recognize preferences to use terms other than power to express the same meaning (such as empowerment; or people, social, or interpersonal skills), but the important point is to move away from the term “soft,” which diminishes the value of these skills and degrades their importance. More than a professional competency, relational care is also the bridge between nurses and the public, restoring trust when health systems feel impersonal and fractured. As evidence shows, relational excellence is foundational to patient and organizational outcomes, not a luxury.^5^

## THE CHALLENGES

In our increasingly transactional and technologically mediated world, patients and families yearn for human presence, often pleading: “Show me you care before you show me how competent you are.” The cataclysmic rise of artificial intelligence (AI) and reliance on technology leave patients and caregivers relishing compassionate engagement with a human versus a series disembodied screens or rote responses.

Nurses know this reality deeply. Reports like the R^3^ Initiative confirm that relationships—between patients and nurses, among colleagues, and between staff and leaders—are the missing ingredient in workplaces strained by burnout, turnover, and disconnection.^6^ At the same time, escalating reliance on AI, institutional pressures, and workplace violence threaten to further erode relational care.

If nursing doesn't claim the essential nature of these skills, we risk allowing them to become expendable. Reframing so-called soft skills is both a professional imperative and an ethical necessity. This reframing is consistent with calls from business and other disciplines to recognize these relational skills as foundational for effective leadership and essential for patient outcomes and workforce sustainability.^1^ In a period marked by deep societal fissures, the trust bestowed on the nursing profession becomes vital fuel for reclaiming the necessity of these skills and elevating their importance.

## CONNECTIVE LABOR AND POWER SKILLS

Allison Pugh, a sociologist, describes the undervalued and invisible role of connective labor in the delivery of health care. What nurses do every day is a form of connective labor—the invisible, emotional, and relational work that sustains belonging and trust in fragmented contexts such as health care. In *The Last Human Job*, Pugh characterizes connective labor as: “Work that relies on empathy, the spontaneity of human contact, and a mutual recognition of each other's humanity.” Inherent in connective labor are the so-called soft skills that we propose to reframe. In nursing, connective labor is a core—though often undervalued—dimension of the profession.^7^ Capacities such as attuning to others' experiences with empathy, authentic listening, perceiving the essence of another, remaining present despite emotional upset or conflict, and staying true to our moral compass when challenged are central to connective labor. By embodying power skills, nurses create conditions for healing, resilience, and ethical practice, even in morally complex or high-pressure environments.

## POWER SKILLS IN ACTION

Nurse leaders in education and practice have extensive opportunities to assert and demonstrate the importance and centrality of power skills to leaders' effectiveness and to their ability to advance these skills within organizations, teams, or individuals. Relational skills redefine leadership. They enable nurse leaders to transform conflict into collaboration, disconnection into trust, and transactional encounters into relational partnerships. The examples below illustrate the centrality of power skills in nurse leaders' repertoire.

### Tom's story: Reframing a policy meeting

Tom, a nurse executive at a major health system, was appointed to a national advisory committee on patient safety. As the only nurse at the table, he observed the first meeting dissolving into competing agendas rather than collaborative dialogue. Drawing on active listening and respectful communication, Tom intervened: “Mr. Chairman, I'm hearing lots of great ideas among the committee. I'm wondering if it would be helpful if I wrote some of these ideas down on the flip chart so we don't lose them, and we can build on them.”

That simple act shifted the dynamic from fragmentation to collaboration. Tom's ability to recognize what was missing, invite connection, and create structure elevated his influence and transformed the group into a trusted, engaged team.

### Martine's story: Navigating conflict with families

Martine, a nurse manager on a busy medical step-down unit, faced escalating tensions with a patient's family after repeated miscommunication with the care team. Over the weekend, security had escorted the patient's son off the unit after an aggressive outburst. On Monday morning, Martine met with the family. Instead of reacting defensively, Martine listened with empathy and suspended judgment.

Through authentic listening, Martine learned about the family's recent losses and their fear that their loved one's care was being compromised. By staying grounded, respectful, and morally centered—even in the face of aggression—Martine moved the situation from conflict toward cooperation. Their presence exemplified how power skills foster trust and restore dignity in high-stakes encounters.

### Maria's story: Supporting a student in clinical practice

Maria, a nursing faculty member supervising students in a clinical rotation, noticed that one of her students, James, was struggling with communication and confidence when interacting with patients. His technical skills were adequate, but he often avoided difficult conversations, leaving patients feeling uncertain and anxious. Instead of focusing solely on his performance gaps, Maria invited James into a reflective dialogue. She asked him what he noticed about his interactions and what emotions he experienced in those moments.

By listening without judgment and validating his concerns, Maria helped James recognize that his avoidance stemmed from fear of “saying the wrong thing.” Together, they practiced simple phrases to acknowledge patients' concerns and built strategies for James to stay present even when uncomfortable. Over time, his confidence and patient engagement improved. Maria's use of empathy, authentic feedback, and scaffolding turned a potentially discouraging situation into a growth opportunity—modeling the very power skills that James was learning to embody.

### Redefining leadership in everyday actions

These three vignettes illustrate how power skills show up in the daily work of nurse leaders, whether at the level of interprofessional leadership (Terri), clinical leadership (Martine), or education (Maria). In each case, leadership emerged not from authority alone, but from the ability to listen deeply, communicate authentically, regulate emotions under pressure, and remain anchored to core values.

## POWER SKILLS AND WORK ENVIRONMENT

Although the importance of individual-level power skills can't be overstated at any stage of a nurse's professional journey, organizations that employ nurses must also evolve to create environments conducive for full deployment of these abilities. Hence, further development of systems-level skills is essential for nurse leaders. To enable connective labor to thrive and workplaces to evolve toward power skills–centered cultures, senior leaders should hone reasoning skills—such as systems thinking and design thinking—to create environments where power skills can flourish.^8^ When these skills are combined with individual power skills, nurse leaders are equipped with a comprehensive toolbox of resources that enable effective advocacy and results.

A CNO faced with staffing shortages within their facility must evaluate the entire upstream system contributions resulting in this outcome, not merely the closest elements within their direct control. Focusing solely on improving the immediate work environment, for instance, may not yield the desired staffing levels when the overall workforce supply in the facility's geographic area doesn't meet the entirety of the demand. Moreover, this type of reductionist thinking rarely yields sustained solutions to systemwide problems and further perpetuates the inability of frontline caregivers to fully deploy connective labor and leverage their power skills.

A thorough investigation of the entire system may reveal to this CNO that upstream nursing workforce supply constraint lies in the design of their state's nursing education policy. Subsequently, this CNO can employ their power skills to engage in regulatory and legislative advocacy and build coalitions aimed at driving changes and impacting the long-term health of both their workplace and the wider community. Conversely, if the CNO decides to improve the workplace culture, they could achieve greater success by using design thinking, a process of creating solutions centered on fully understanding and meeting the needs of those for whom these solutions are intended.^9^ In this instance, the CNO could engage in design thinking to build a workplace that fully addresses and supports the staff's multidimensional wellness needs, encompassing, among others, emotional, financial, occupational, social, and moral aspects, hence also creating a culture conducive to the full engagement of power skills at all levels.

For nurse leaders, power skills and systems skills aren't abstract ideals; they're lived practices that shape meetings, conversations, teaching moments, and crises responses every day. They're the invisible infrastructure that enables nurses to influence systems, protect patients, and cultivate the next generation of nurses.

### Shifting the narrative

The disempowering language of soft skills has been partly imposed from outside, but nurses have also internalized and perpetuated this messaging. The words we use shape the way the profession is valued. We sustain these narratives through the words, phrases, and messages we continue to consciously or unconsciously repeat. These narratives have the power to either provoke fear and disconnection, or connection, community, and understanding. Reframing soft skills isn't about creating hierarchies among competencies—it's about claiming the rightful place of relational expertise alongside technical proficiency. For leaders, amplifying individual power skills with systemic power skills, like design and systems thinking, creates sustainable ecosystems for nursing practice and education.

Power skills are core competencies that help reduce burnout, strengthen trust, and build equitable, person-centered care. Education and practice must reflect this goal. Too often, technical checklists dominate while relational competence is undermeasured, undertaught, and undervalued. Elevating power skills, as well as design and systems thinking, means embedding these skills in curricula, onboarding, and professional development, and valuing them as highly as clinical and technical expertise. When nurses adopt this mindset during their education, they're more likely to embody this perspective in practice.

Shifting the narrative also means aligning nurses with the public. Individuals, families, and communities value being seen, heard, and respected. Recognizing power skills elevates nursing as a collaborative force with communities, advocating for care that's rooted in connection, dignity, and accountability.

### A call to action

Now is the time. Communities need nurses not only for practice proficiency but for the healing power we bring. We must reclaim relational care as a professional and social cornerstone.

We call on nurse leaders, educators, policymakers, and every nursing professional and organization to:

***1. Embrace the value of relational care***. No longer can we afford to apologize for or disvalue the fundamental and vital role of nurse's relational care. Seek opportunities to elevate the importance of relational skills in all encounters and illustrate their centrality to healing, equity, safety, and trust.
***2. Reframe our vocabulary***. Abandoning the use of soft skills—and embracing capacities of trust, resilience, and moral engagement—offers an essential scaffolding for elevating the value of nurses' contributions to health and health care.
***3. Model and embody power skills***. Through words and actions, demonstrate how cultivating power skills and a caring presence bolsters outcomes, team cohesion, and nurse well-being. Nurse leaders and educators have a pivotal role in creating cultures in which students and practicing nurses are consistently exposed to role models who embrace and value power skills.
***4. Embed power skills structurally***. Integrate power skills in onboarding, education, evaluations, and competency frameworks in nursing education and practice. Begin integration from the first day of nursing school, in transition into practice, and throughout the nursing career. Nurse leaders can model these skills within schools, practice settings, and professional organizations as they implement systems and design methods to address systemic issues and create sustainable results.
***5. Advocate for systemic recognition***. Leverage professional recognition and funding priorities, develop policies that proportionately value relational outcomes with technical quality, and partner with media and communications to advance a new vocabulary and story about the value of nursing.^10^
***6. Share new narratives***. By sharing stories like those of Tom, Martine, and Maria—leaders can illustrate how relational presence transforms care, learning, and leadership.
***7. Align with the public***. Cocreate a narrative of care, fairness, and integrity-preserving action with patients and communities. When nurses and the public stand together, power skills become a collective force for healing health care and society.

## NAME IT AND CLAIM IT

Nursing's impact and value extend beyond technical expertise and reside in nurses' capacity for connection and systemic reasoning. At a time when disconnection and mistrust threaten the very fabric of care—and regulatory, financial, education, and employment systems are failing—power skills are the foundation for reclaiming nursing's voice, authority, and future. The opportunity before us is clear: either continue to allow these skills to be dismissed as soft or boldly assert their rightful place as essential tools for healing, leadership, and transformation.

Now is the time for nurses to rewrite the narrative, to elevate the skills that have always defined the profession and to stand together in affirming that relational power is nursing's greatest strength. By acting now, we can reclaim our authority and reassert that nursing's greatest contribution lies not only in what we do, but in how we connect, protect, and lead through relationships.
